# COGNITIVE FUNCTIONING AMONG BREAST CANCER SURVIVORS AND NON-CANCER PARTICIPANTS: EVIDENCE FOR SIMILARITIES

**DOI:** 10.1093/geroni/igz038.2411

**Published:** 2019-11-08

**Authors:** Giancarlo Pasquini, Brent J Small, Jacqueline Mogle, Martin Sliwinski, Stacey B Scott

**Affiliations:** 1 Stony Brook University, Stony Brook, New York, United States; 2 University of South Florida, Tampa, Florida, United States; 3 Penn State University, University Park, Pennsylvania, United States; 4 Penn State University, Center for Healthy Aging, University Park, Pennsylvania, United States

## Abstract

Breast cancer survivors may experience accelerated decline in cognitive functioning compared to same-aged peers with no cancer history (Small et al., 2015). Survivors may show important differences in mean-level performance or variability in cognitive functioning compared to those without a history of cancer (Yao et al., 2016). This study compared ambulatory cognitive functioning in a sample of breast cancer survivors and an age-matched community sample without a history of cancer (n_cancer=47, n_non-cancer=105, age range: 40-64 years, M=52.13 years). Participants completed three cognitive tasks measuring working memory, executive functioning, and processing speed up to five times per day for 14 days. Results indicated no mean-level differences in cognitive performance on the three tasks between cancer survivors and those without cancer history (p’s>.05). Unexpectedly, women without cancer history showed more variability than survivors on working memory but not on the other two tasks. Across both groups, those without a college education performed worse on executive functioning (B=-0.05, SE=0.03, p<.05) and working memory (B=0.94, SE=0.36, p<.05) compared to those that completed college. Additionally, older age was associated with slower processing speed (B=31.67, SE=7.44, p<.001). In sum, this study did not find mean-level group differences in cognitive functioning between cancer survivors and age-matched women without a history of cancer. Contrary to hypotheses, those without a history of cancer were more variable on working memory. Results suggested similarities in cognitive functioning in the two samples and that education and age are important predictors of cognitive functioning independent of cancer history.

